# Cell size, genome size, and maximum growth rate are near‐independent dimensions of ecological variation across bacteria and archaea

**DOI:** 10.1002/ece3.7290

**Published:** 2021-03-16

**Authors:** Mark Westoby, Daniel Aagren Nielsen, Michael R. Gillings, Elena Litchman, Joshua S. Madin, Ian T. Paulsen, Sasha G. Tetu

**Affiliations:** ^1^ Department of Biological Sciences Macquarie University Sydney NSW Australia; ^2^ Kellogg Biological Station Michigan State University Hickory Corners MI USA; ^3^ Hawaii Institute of Marine Biology University of Hawaii Kaneohe HI USA; ^4^ Department of Molecular Sciences Macquarie University Sydney NSW Australia

**Keywords:** archaea, bacteria, cell diameter, ecological strategies, genome size, maximum growth rate, traits

## Abstract

Among bacteria and archaea, maximum relative growth rate, cell diameter, and genome size are widely regarded as important influences on ecological strategy. Via the most extensive data compilation so far for these traits across all clades and habitats, we ask whether they are correlated and if so how. Overall, we found little correlation among them, indicating they should be considered as independent dimensions of ecological variation. Nor was correlation evident within particular habitat types. A weak nonlinearity (6% of variance) was found whereby high maximum growth rates (temperature‐adjusted) tended to occur in the midrange of cell diameters. Species identified in the literature as oligotrophs or copiotrophs were clearly separated on the dimension of maximum growth rate, but not on the dimensions of genome size or cell diameter.

## Graphical abstract

Among bacteria and archaea, maximum relative growth rate, cell diameter, and genome size are widely regarded as important influences on ecological strategy. Via the most extensive data compilation so far for these traits across all clades and habitats, we found little correlation among them, indicating they should be considered as independent dimensions of ecological variation.

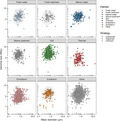

## 1 INTRODUCTION

**FIGURE A1:**
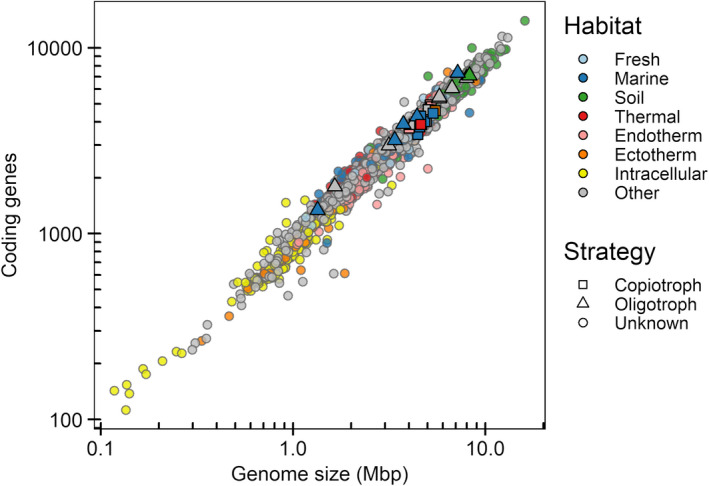
Relationship across species between number of different coding genes and total genome size. Ordinary least squares regression has *r* ^2^ of .976 across 3,300 species. In the habitat classification (color scheme), fresh and marine waters include both water and sediment. Intracellular species are those making a living inside eukaryote cells. Host‐associated species are attributed to endotherm or to ectotherm hosts if they multiply within the host body or gut, or to “other” if they grow on the host’s external surface or are associated with plants, algae, or fungi. Species without habitat information are also attributed to “other.” Species identified in the literature as copiotrophs or oligotrophs (Table A1) are denoted by squares and triangles, respectively

**FIGURE A2:**
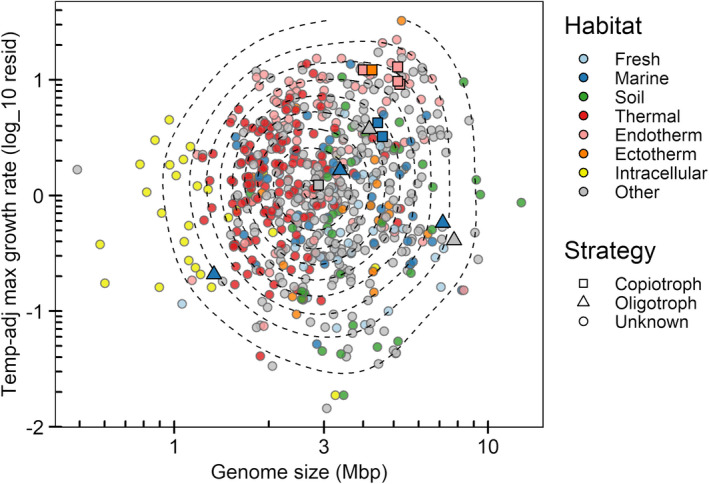
Temperature‐adjusted maximum growth rate in relation to genome size across species, including intracellular species. 646 species, *r* ^2^ = .0066. Dotted lines are density contours. Habitat and copiotrophy–oligotrophy coding as in Figure A1

**FIGURE A3:**
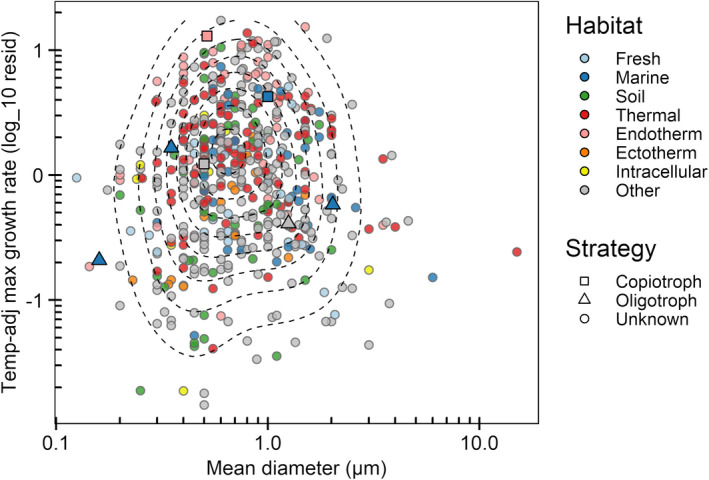
Temperature‐adjusted maximum growth rate in relation to cell radial diameter across species, including intracellular species. 529 species, *r* ^2^ = .00044. Habitat and copiotrophy–oligotrophy coding as in Figure A1

**FIGURE A4:**
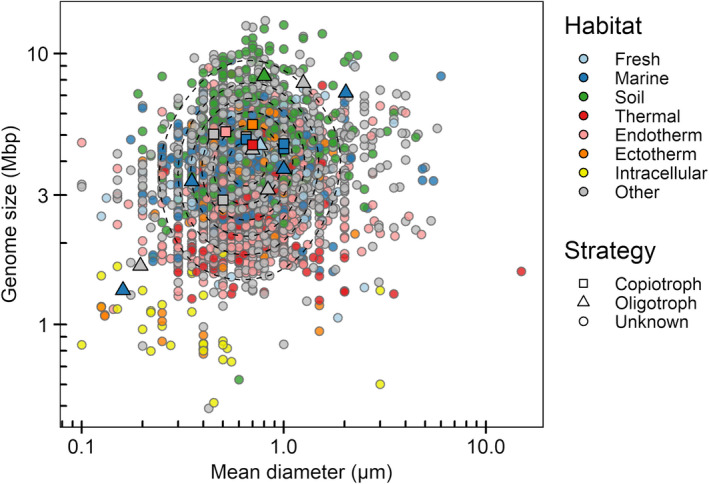
Genome size in relation to cell radial diameter across species, including intracellular species. 3,502 species, *r* ^2^ = .019. Habitat and copiotrophy–oligotrophy coding as in Figure A1

To the extent ecological strategies of species can be captured via measurable traits, this makes comparisons possible at global scale. For vascular plants on land, major dimensions of strategy variation have been described through traits (e.g., Díaz et al., 2016), and responses to competition have been generalized across different vegetation types through traits (e.g., Kunstler et al., 2016). The possibility of a trait‐based ecology for bacteria has been advocated by several research groups (Fierer et al., 2007, 2014; Hall et al., 2018; Ho et al., 2017; Krause et al., 2014; Litchman et al., 2015; Litchman & Klausmeier, 2008; Malik et al., 2020; Wood et al., 2018), but up to the present has taken the form of discussing concepts or interpreting particular study situations. Based on synthesis of quantitative and phenotypic trait data across bacteria and archaea as a whole (Madin et al., 2020), we here assess correlation patterns among some major traits and consider what they imply for ecological strategies. By “as a whole,” we mean spanning all clades and habitats, but excluding species that have not been brought into culture. For species known only from metagenomic assembly, cell sizes and maximum growth rates are not known; hence, they are not included. It is possible that trait correlation patterns among not‐yet‐cultured species may prove different, but since phenotypic trait data are not yet available for them, that question cannot be addressed at present. For the main‐text narrative, we have excluded also mycoplasmas and other taxa specialized to make their living inside eukaryote cells. Versions including these taxa are shown in Appendix Figures A1, A2, A3, A4.

In the present paper, we focus on cell diameter, genome size, and maximum growth rate. These traits are widely thought to have important roles in ecological strategy among bacteria and archaea (reviewed briefly below), and they are available across a reasonably wide range of species. Major habitat groups are considered as a potential influence. Relationships to aerobic versus anaerobic metabolism are discussed elsewhere (Nielsen et al., 2021). The question addressed here is how these three quantitative traits correlate with each other across species. Consider the following two ends of a spectrum of possibilities. At one end, the three traits might vary independently, meaning that at any given level for one, a wide range of values for the others can be found. This might be expected on the basis that each is capable of evolving independently of the others. At the other end of a spectrum of possibilities, all these traits might be coordinated with the oligotrophy–copiotrophy spectrum generally regarded as important in bacterial ecology. If oligotrophy favors small cells, small genomes and slow maximum growth rate, and if the oligotrophy–copiotrophy spectrum is a major influence on variation across species, then we would expect all these traits to be distinctly correlated across species. Further if such a correlation were present, then a subsidiary question would be whether it was clearly evident within habitats, or whether it might take the form mainly of differences between relatively oligotrophic habitats such as pelagic water versus relatively copiotrophic habitats such as waste water.

We first summarize briefly what is known about each of the three quantitative traits, then turn to their relationships to copiotrophy and oligotrophy.

### 1.1 Cell size

Recorded mean cell radial diameter varies about one order of magnitude across species, running mostly between about 0.2 and 3 μm. Cell volume varies more widely, being the cube of a linear dimension and also due to the diversity of cell morphologies. Here, we adopt radial diameter as our main descriptor of size. It captures surface area to volume relations effectively both for spheroidal cocci and for rod‐shaped bacilli, the two most common shapes.

Potential diffusion of substrate toward and into the cell, per cell volume, increases steeply as cells become smaller, to −2 power of radius (Fenchel et al., 2012; Fiksen et al., 2013; Jumars, 1993; Madsen, 2008). This means that smaller cells can sustain a given consumption rate per cell volume from lower ambient substrate concentrations. It has been seen as a reason why small cells should be favored in oligotrophic settings (e.g., Madsen, 2008; Schulz & Jorgensen, 2001).

Lower limits to cell diameter are thought to be set by costs of cell wall and membrane construction becoming larger at the expense of investment in synthetic and metabolic machinery. For example, a calculation by Raven (1994) suggested that boundary membranes reach more than 30% of cell dry mass by the time a spherical cell becomes as small as 0.5 μm radius.

Cell sizes are known to adjust plastically within cell lineages in response to substrate supply (Lever et al., 2015), with volumes decreasing up to 10‐fold after 28 days of starvation conditions compared with growth conditions. Available cell size measurements have nearly all been made under laboratory growth conditions. Measurements can be considered standardized in this respect, and should capture differences across species, though not necessarily reflecting actual field cell sizes.

### 1.2 Genome size

Variation in genome size across bacteria and archaea reflects mainly the number of different coding genes, rather than noncoding sequence or genes found in multiple copies (Konstantinidis & Tiedje, 2004; and this was true in our dataset also, Figure A1). Genome size can therefore be thought of as capturing ecological strategy variation along a versatility dimension (Guieysse & Wuertz, 2012). It is expected to reflect the range of different resources that can be transported or metabolized, together with flexibility in responses to different circumstances. Consistent with this interpretation, genome size is correlated with the proportion of the genome occupied with receiving internal and external signals and using those to modify gene expression, and also with aerobic metabolism and with sporulation (Nielsen et al., 2021).

Much discussion has focused on genome reduction (Giovannoni et al., 2014; Swan et al., 2013). This takes two disparate forms (Giovannoni et al., 2014). Species that grow inside eukaryote cells or otherwise in very intimate association often have come to rely on their associate to provide metabolic products and the corresponding pathways are no longer present in their own genome. Small effective population sizes increase the importance of drift relative to selection (Bobay & Ochman, 2018), making more genes effectively neutral and prone to be eliminated. In contrast, where effective population sizes are large and resources low, selection can minimize resources required for replication. The pelagic taxa *Prochlorococcus* and *Pelagibacter* are exemplars.

### 1.3 Maximum growth rate

Maximum growth rate is the potential relative rate of increase under favorable growth conditions, *μ*_max_ in the Monod equation. Like measurements for cell size, it should be thought of as a bioassay that captures differences across species, not as a typical field observation. The growth temperatures adopted for culture vary across species and growth rates tend to be faster at higher temperatures. Here, we use a temperature‐adjusted maximum growth rate.

Also of interest, and investigated in appendices, is ribosomal RNA operon copy number (RRN). This is a contributor to maximum growth rate and is quite widely used as a proxy for it (Nemergut et al., 2016; Niederdorfer et al., 2017; Valdivia‐Anistro et al., 2016). However, reported correlations between RRN and maximum growth correspond to only moderate *r* ^2^ values in the range .15–.35 (Nielsen et al., 2021; Vieira‐Silva & Rocha, 2010). Both maximum growth rate and RRN are expected to be most strongly under selection in lifestyles where resources become episodically available and there is a race to convert them into population. For example, Li et al. (2019) showed that RRN was not correlated with growth rates in soil, but became correlated with growth rates following glucose addition.

Larger RRN allows species to build up ribosome numbers faster and perhaps to maintain larger numbers. However, the more ribosomes produced or maintained, the less protein is available for metabolic machinery that would use substrate more completely (Flamholz et al., 2013; Molenaar et al., 2009; Polz & Cordero, 2016; Roller et al., 2016). Accordingly, high RRN is associated with a rate‐yield trade‐off, whereby faster‐multiplying populations are less efficient in converting substrate into cell material (Polz & Cordero, 2016). The rate‐yield trade‐off occurs also as plastic response, with gene expression shifting to economize on possible downstream mechanisms of energy use. In summary, RRN and potential rate of increase are correlated, but not identical.

Overall, enough is known to feel confident that cell size, genome size, maximum growth rate and RRN are each an important influence on the ecology of bacterial and archaeal species.

### 1.4 Traits in relation to the oligotrophy–copiotrophy spectrum

A strategy spectrum widely regarded as important in microbial ecology runs from oligotrophy, coping with low resource supply, to copiotrophy, the capacity to take advantage of rich resource supply (Fenchel et al., 2012; Fierer et al., 2007; Madsen, 2008). This spectrum is expected both on a within‐habitat and a between‐habitat basis. Between habitats, some environments such as deep aquifers and the pelagic waters of central gyres clearly offer much lower levels of resource supply than (say) wastewater treatment plants. Within habitats, opportunity for many heterotrophic bacteria and archaea arises in the form of successions initiated by an injection of substrate, via (say) death of a zooplankter or production of a fecal pellet. Initial occupancy of such a resource is expected to favor copiotrophs that capture a large proportion by rapid multiplication. As resource concentrations become depleted, the competitive balance is expected to shift to oligotrophic taxa that can sustain growth from lower substrate concentrations.

The strongest expectation is that oligotrophs will have slower maximum growth rates than copiotrophs and that these will be associated with higher yields and lower RRN. It has also been quite widely argued that oligotrophy should be characterized by smaller cell sizes (Giovannoni et al., 2014; Lauro et al., 2009; Lever et al., 2015; Poindexter, 1981) and smaller genome sizes (Fierer, 2017; Giovannoni et al., 2014), although Poindexter (1981) reasoned that oligotrophs needed to extract all possible energy from substrate, which would often require them to have multiple pathways and to be aerobic. Some have sought to apply the competitor–stress tolerator–ruderal (CSR) strategy triangle from plant ecology to microbes, with the S dimension of this scheme corresponding to oligotrophy (Fierer, 2017; Krause et al., 2014). These treatments similarly suggest small cell size and small genomes may tend to be associated with oligotrophy.

So then, if these expectations for oligotrophy are correct, and if also the oligotroph to copiotroph spectrum is a substantial influence on variation across bacterial and archaeal species, we would expect to find correlation across species among small cell size, small genome size, slow maximum growth rate, and low RRN. At the other end of the spectrum of possibilities, these traits might vary more or less independently. This would mean that they operated separately as influences on ecological strategy, and all combinations have been able to emerge during the course of prokaryote evolution.

We note that DeLong et al. (2010) and Kempes et al. (2012, 2016) have argued that maximum growth rate, genome size, and cell size are observed to be positively correlated across species. Their data are compared with ours in Appendix B. Briefly, the differences in conclusions trace mainly to which species are included and how many.

## 2 METHODS

The species‐by‐traits dataset used here is produced by a scripted workflow, described in depth by Madin et al. (2020), that reproducibly merges 26 existing datasets. Most records in the datasets are at the level of genotypes or 16S rRNA phylotypes. The workflow (a) prepares datasets to be merged; (b) combines datasets and condenses equivalent traits into columns; and (c) condenses rows into species based on the GTDB taxonomy (Parks et al., 2018) (https://gtdb.ecogenomic.org). This taxonomy applies the conventional criterion of average nucleotide identity ≥96.5% for grouping entities into species.

Where there are multiple records for a species, these are condensed down to a single row. The records are typically averaged (for quantitative traits) or a majority rule is applied (for categorical traits). The rules are specified in more detail below for selected traits and in Madin et al. (2020). During this process, standard deviations have been calculated and outliers identified. A substantial number of records have been corrected, or sometimes removed as not credible. A table of these corrections is implemented by the code. The number of records for individual species ranges from >10,000 for *Staphylococcus aureus* down to 1 for many species. Among the traits considered here, maximum growth rate has the least coverage at 618 species, but this is still an advance over the 214 species in previous compilations (Vieira‐Silva & Rocha, 2010).

Our aim was to develop coverage of traits and their correlations as widely as possible across bacteria and archaea. We have condensed to species level as a working compromise, intended to capture ecologically meaningful variation without letting the dataset be unduly dominated by a few species with thousands of records each (e.g., *Staphylococcus aureus*, *Salmonella enterica*, *Streptococcus pneumoniae*). Because our focus has been on phenotypic traits such as cell diameter and potential rate of increase, the data come largely from species that have been brought into culture. These may tend to have larger genomes and faster potential growth rates and more often to be aerobic, compared with the many uncultured species (Fierer, 2017; Giovannoni et al., 2014; Nayfach & Pollard, 2015; Solden et al., 2016). However, the species included here do span a full range of possibilities, including extreme oligotrophy, very small genome sizes, and very slow potential growth rates.

For purposes of the main text, we have excluded species that live inside the cells of eukaryotes, and also mycoplasmas as a group. These are well known to have strongly reduced genomes for reasons not connected to oligotrophy, and their maximum growth rates must be conditioned by relations with their host as well as by their uptake and conversion of resources. There were 35 such species in our dataset with both genome size and cell diameter, and 27 such species with both genome size and maximum growth rate. They are included in Figures A1, A2, A3.

We have built a list of species (Table A1) identified in the literature as definite oligotrophs or definite copiotrophs, in order to be able to position these in the trait‐space figures. To avoid circularity, we have not applied criteria of our own to the question whether they are oligotrophs or copiotrophs, but have adopted the opinions of the authors of the papers.

Because maximum growth rates tend to be faster for species cultured at higher growth temperatures, we have used here temperature‐adjusted maximum growth rates, which are residuals from the regression fit log_10_(max growth) = 0.0105(growth temp) − 1.2003, *r* ^2^ = .11. In other words, these are deviations above or below the expected mean max growth at their growth temperature, in log_10_ units. The basis for adopting this particular temperature adjustment is explained further at Appendix C.

The data reported here are survey or correlative. As is well known, correlation unlike manipulative experiments cannot prove causation, because of the likelihood of cross‐correlation with other variables, including those unmeasured and unconsidered. Accordingly, the statistics presented should be interpreted as quantifying variation and correlation across species, rather than as significance tests of hypotheses about causation. For the major correlations, we provide also versions partialled for phylogeny, using phylogenetic generalized least squares (PGLS) via phylolm (Tung Ho & Ané, 2014). Phylolm v2.6.1 was installed from https://github.com/lamho86/phylolm. The phylogenetic tree adopted corresponded to GTDB taxonomy with seven levels (superkingdom, phylum, class, order, family, genus, and species), star phylogeny at each node, and unit branch lengths. GTDB taxonomy was adopted because it is monophyletic, so far as can be determined from the 120 protein‐coding genes used, and because it places taxonomic ranks at a consistent relative distance from the tree root. Partialling for phylogeny via PGLS has the effect of measuring correlation of trait divergences averaged across the ensemble of nodes. Compared to correlation across present‐day species, it downweights differences between major clades.

## 3 RESULTS

**FIGURE 1:**
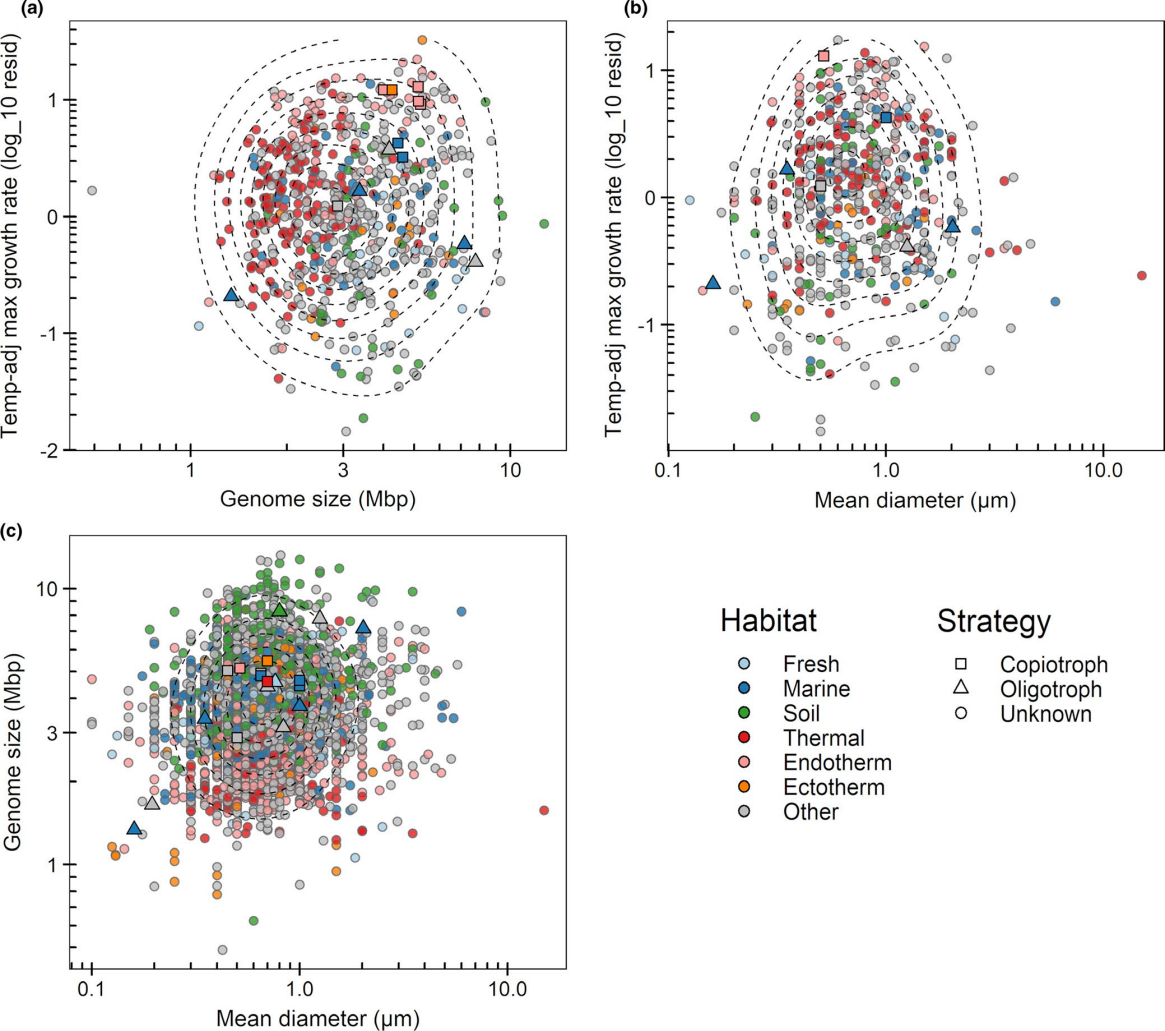
(a) Temperature‐adjusted maximum growth rate in relation to genome size across species. (b) Temperature‐adjusted maximum growth rate in relation to cell radial diameter across species. (c) Genome size in relation to cell radial diameter. Dashed lines indicate density contours. In the habitat classification (color scheme), fresh and marine waters include both water and sediment. Host‐associated species are attributed to endotherm or to ectotherm hosts if they multiply within the host body or gut, or to “other” if they grow on the host's external surface or are associated with plants, algae, or fungi or have no habitat attributed. Species identified in the literature (Table A1) as copiotrophs or oligotrophs are denoted by squares and triangles, respectively

Across culturable species where records are available, there was little to no correlation (2% of variation or less) among temperature‐adjusted maximum growth rate, cell radial diameter, and genome size (*r* ^2^ values in Table 1, Figure 1a–c). The same was true of correlations partialled for phylogeny (Table A2).

**TABLE 1:** Correlation *r* ^2^ among the four traits considered here, all log‐scaled. Number of species for each trait pair given next to the correlation

Trait	Genome size	Cell radial diameter	rRNA operon copy number
Temperature‐adjusted max growth rate	.00425 (*n* = 618)	.000456 (*n* = 519)	.303 (*n* = 388)
Genome size		.0121 (*n* = 3,466)	.138 (*n* = 2,726)
Cell radial diameter			.0225 (*n* = 925)

**FIGURE 2:**
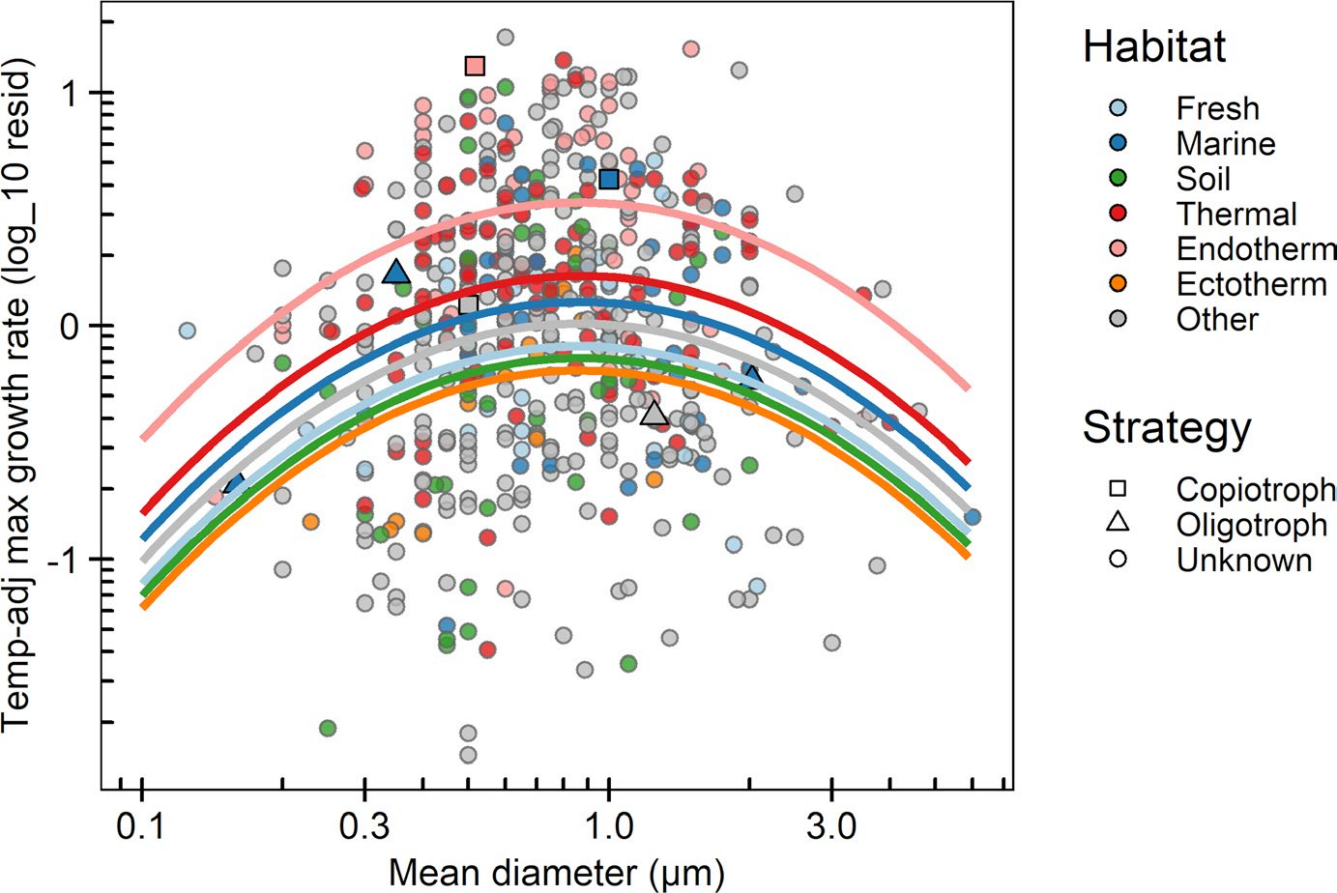
Temperature‐adjusted maximum growth rate in relation to cell diameter, with polynomial fits separated by habitat. The model (Table 2) accounts for about 17% of variance in log temp‐adjusted maximum growth rate in total, with habitat contributing about 11% and nonlinear response to cell diameter about 6%. Coefficients of the model are in Table A3. While the models in Table 2 use only species for which all data are available so that AIC is comparable, use of all available species for each model (Table A4) shows similar patterns

Although there was little overall correlation between maximum growth rate and cell radial diameter, there was some evidence for a particular nonlinearity, with the fastest growth rates tending to occur in the midrange of cell diameters (Figure 2). If indeed lower and upper limits to cell size coincide with disadvantage, at the small‐diameter end from increasing relative allocation to cell envelope, and at the large‐diameter end from decreasing diffusive uptake per cell volume, it would make sense that very fast growth rates were only achievable in the midrange of sizes. Note, however, that slow maximum growth rates were also common in the midrange of cell sizes.

A more complete search for interactions or nonlinearities is described in Table 2. The most substantial contributions to *R* ^2^ were for a nonlinear response to cell diameter (model 4 in Table 2, *ca*. 6%) and for habitat (model 6, *ca*. 10%). The best model overall by AIC (model 7) simply had these two effects additive, and *R* ^2^ = 0.167. This is the model fitted in Figure 2. Providing for interaction between the response to diameter and habitat (model 8) and for interactions of all these with genome size (model 9) did not increase *R* ^2^ commensurate with the *df* invoked, and AIC deteriorated.

**TABLE 2:** Models with successively more terms for predicting temperature‐adjusted maximum growth rate, showing AIC, multiple *R* ^2^, and associated *df*

Model	AIC	*R* ^2^	*df*
1. growth ~ cell_diam	700.69	0.000893	384
2. growth ~ genome_size	700.99	0.000099	384
3. growth ~ cell_diam * genome_size	698.95	0.0156	382
4. growth ~ cell_diam + cell_diam^2	679.13	0.0600	383
5. growth ~ genome_size + genome_size^2	702.85	0.000474	383
6. growth ~ habitat	667.98	0.106	379
7. growth ~ (cell_diam + cell_diam^2) + habitat	644.29	0.167	377
8. growth ~ (cell_diam + cell_diam^2) * habitat	654.69	0.196	365
9. growth ~ (cell_diam + cell_diam^2) * genome_size * habitat	679.32	0.232	344

Note

All quantitative variables were log_10_ scaled. In the model notation, the terms after ~ show what predictors are offered, and * instead of + indicates interactions were provided for, as well as additive terms. Models shown here use only data rows for which all variables are available, so that the AIC values are commensurate. Table S3 shows the same models fitted to all available data for each model.

**FIGURE 3:**
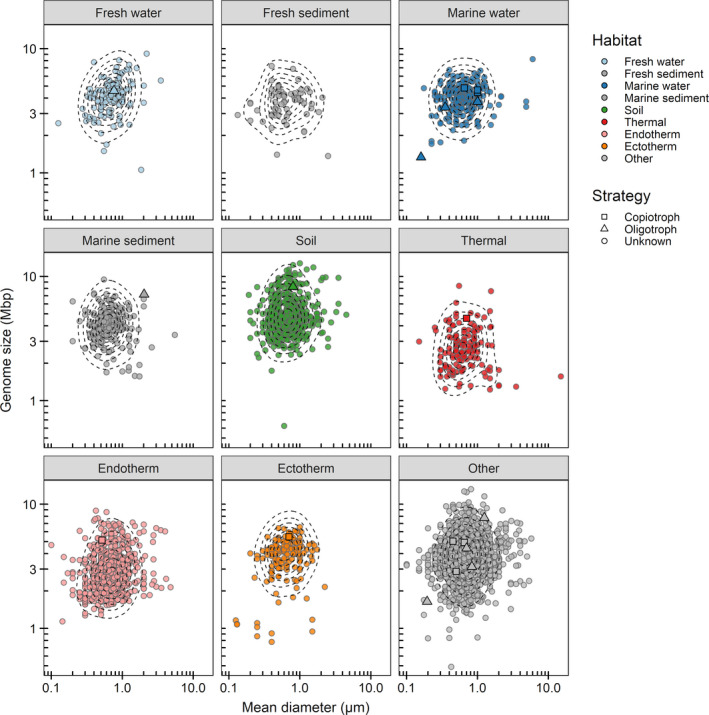
Genome size in relation to cell radial diameter, separated by habitat type. Symbols as in Figure 1

Other points of interest in Figure 1, besides the absence of substantial correlation across species, are as follows. First, correlation was absent also within major habitat types (color scheme in Figure 1, and the cell size–genome size graph further separated into habitats in Figure 3). There was no indication of oligotrophy‐related correlations within particular habitats such as marine waters, with these then being obviated by differences between different major habitats. Second, certain species are indicated that have been explicitly identified in the literature as either oligotrophs (triangle symbols) or copiotrophs (square symbols) (listed in Table A1). These were rather clearly separated on the dimension of maximum growth rate, but not on the dimensions of genome size or cell radial diameter. Third, species from thermal environments tended to smaller genome sizes (Figure 1a,b), as observed previously (Lear et al., 2017; Sabath et al., 2013; Sauer & Wang, 2019; Sorensen et al., 2019). Fourth, the density contours in Figures 1 and 3 were more or less circular. This indicates little interaction between the two traits. The corners of the trait space are not unachievable, but are thinly occupied simply because of low incidence in each dimension.

**FIGURE A5:**
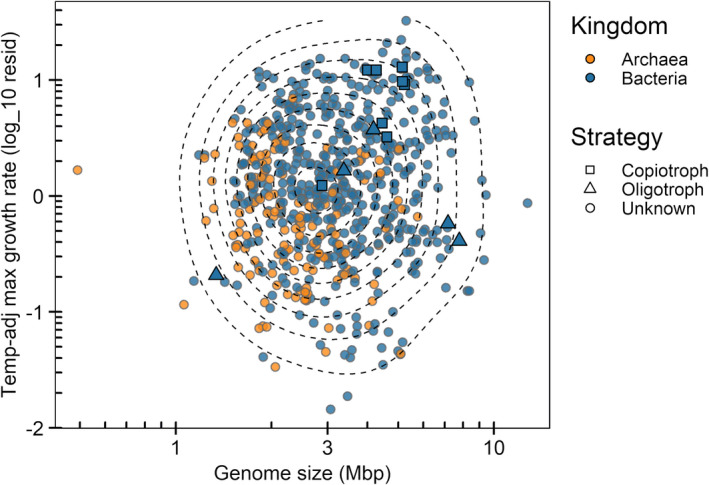
Temperature‐adjusted maximum growth rate in relation to genome size across species, showing archaea vs bacteria and excluding intracellular species. Species identified in the literature (Table A1) as copiotrophs or oligotrophs are denoted by squares and triangles respectively

**FIGURE A6:**
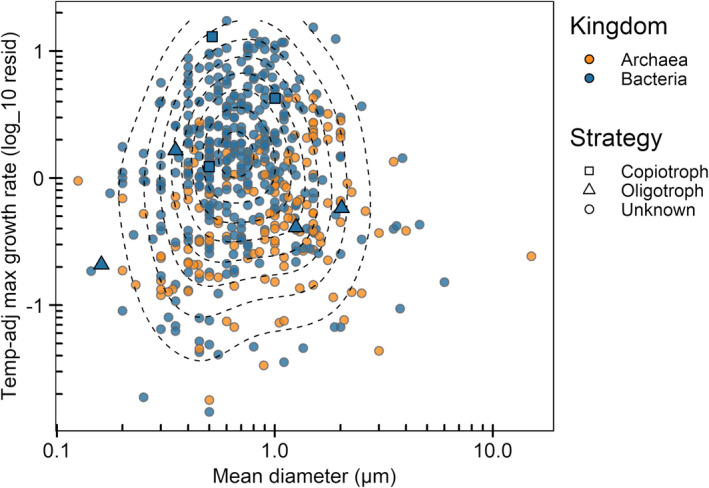
Temperature‐adjusted maximum growth rate in relation to mean cell radial diameter across species, distinguishing archaea from bacteria

**FIGURE A7:**
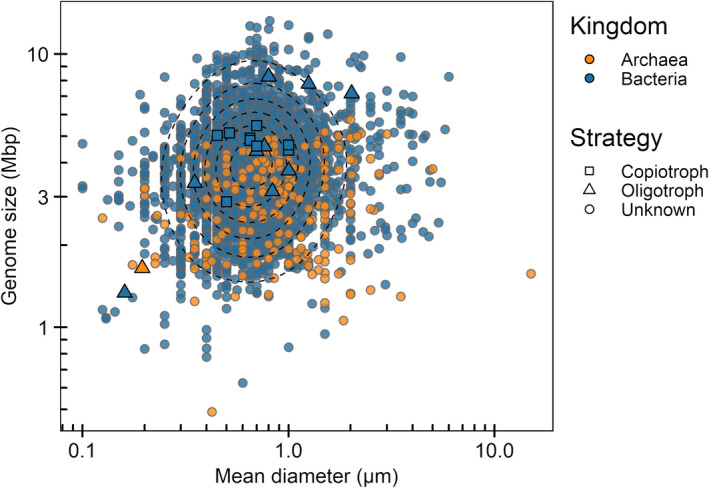
Genome size in relation to mean cell radial diameter across species, distinguishing archaea from bacteria

The independent variation among maximum growth rate, genome size, and cell diameter was not much affected by including species that make a living within eukaryote cells (Figures A2, A3, A4; discussed further in Appendix B). Archaea tended to smaller genomes than bacteria, but correlation was equally absent within each domain (Figures A5, A6, A7).

**FIGURE A8:**
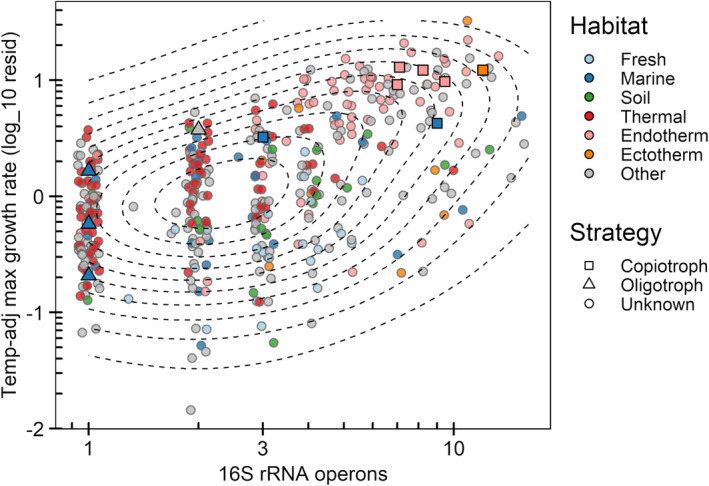
Relationship across species between ribosomal RNA operon copy number and temperature‐adjusted maximum growth rate; *r* ^2^ = .30 across 389 species. rRNA operon counts have been averaged across multiple records within species, where available, hence noninteger counts sometimes appear. Host‐associated species were attributed to endotherm or ectotherm hosts, or to “other” if they came from external animal surface or were associated with plants, algae, or fungi, or had no habitat attributed. Species identified in the literature (Table A1) as copiotrophs or oligotrophs are denoted by squares and triangles, respectively

**FIGURE A9:**
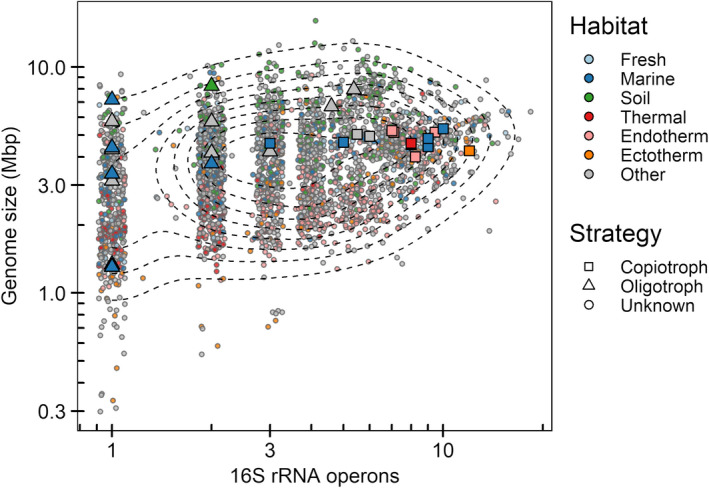
Genome size in relation to rRNA operon copy number; *r* ^2^ = .14 across 2,727 species, or if those with growth temperature >50°C are excluded, *r* ^2^ = .048 across 1,666 species. In the habitat classification (color scheme), fresh and marine waters include both water and sediment. Host‐associated species are attributed to endotherm or to ectotherm hosts if they multiply within the host body or gut, or to “other” if they grow on the host’s external surface or are associated with plants, algae, or fungi, or have no habitat attributed. Species identified in the literature (Table A1) as copiotrophs or oligotrophs are denoted by squares and triangles, respectively

**FIGURE A10:**
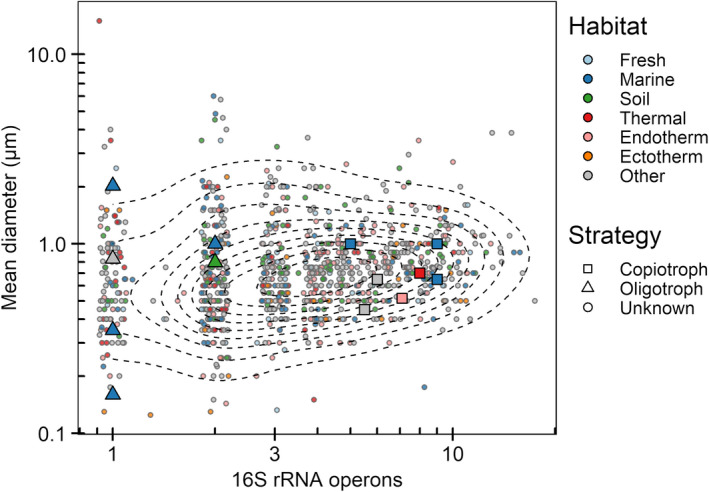
Cell radial diameter in relation to rRNA operon copy number; *r* ^2^ = .023 across 926 species. In the habitat classification (color scheme), fresh and marine waters include both water and sediment. Host‐associated species are attributed to endotherm or to ectotherm hosts if they multiply within the host body or gut, or to “other” if they grow on the host’s external surface or are associated with plants, algae, or fungi, or are not attributed to any habitat. Species identified in the literature (Table A1) as copiotrophs or oligotrophs are denoted by squares and triangles, respectively

Ribosomal RNA operon copy number RRN was indeed correlated with temperature‐adjusted maximum growth rate (Table 1, Figure A8), as expected and as previously shown from smaller datasets without temperature adjustment (Klappenbach et al., 2000; Vieira‐Silva & Rocha, 2010). RRN was also correlated with genome size (Table 1, Figure A9), with large RRN not being found in association with small genome sizes. Species identified in the literature as copiotrophs (squares in Figures A8 and A9) rather consistently had higher RRN than identified oligotrophs (triangles in the figures), as they did faster temperature‐adjusted maximum growth rates. RRN is also a quantity that is available across more species than maximum growth rates. However, RRN, like maximum growth rate, was hardly correlated with cell radial diameter (Figure A10).

## 4 DISCUSSION

### 4.1 Individual relationships

Although discussion of genome reduction often assumes that shedding genes will be an advantage unless they confer some definite benefit, it has been known for some time that maximum growth rate is not faster in species with smaller genomes (Vieira‐Silva & Rocha, 2010). Figure 1 confirms this result with expanded coverage. This is possible because fast‐doubling species commonly operate more than one set of bidirectional replication forks at the same time (Vieira‐Silva & Rocha, 2010). This in turn has consequences for genome architecture. Genes closer to the origin are expressed in more copies at any given time, and it appears that genes are rearranged so that these distance‐dosage effects are beneficial, particularly for genes coding for rRNA, RNA polymerase, ribosomal protein, tRNA, and ubi‐tRNA. There are advantages to high expression of these genes during rapid growth.

**FIGURE A11:**
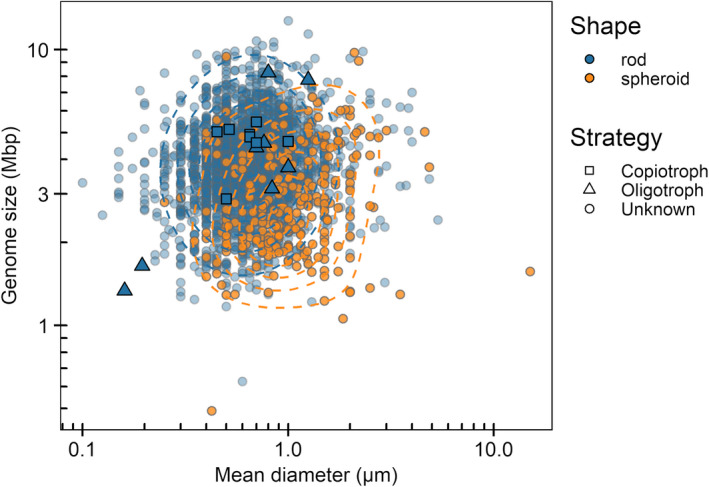
Mean cell radial diameter in relation to genome size, separating rod‐shaped bacilli from near‐spheroidal cocci and coccobacilli. Species identified in the literature (Table A1) as copiotrophs and oligotrophs are denoted by squares and triangles, respectively

The absence of correlation between genome size and cell radial diameter implies either that there is little consistent relationship between the mass of cell machinery and the radial diameter (in other words larger‐diameter species tend to have lower‐density cytoplasm), or that there is little relationship between the genome size and the mass of cell machinery, or both of those things. Rod‐shaped bacteria tended to have slightly larger genomes and slightly smaller radial diameters than spheroidal (Figure A11), but with little correlation evident within either shape.

### 4.2 Overall conclusions

The principal result emerging has been that genome size and cell radial diameter vary across species substantially independently from each other and from temperature‐adjusted maximum growth rate and RRN. A wrinkle on this is that it appears especially rapid growth rates are not found at the upper and lower edges of the cell diameter range. At the lower edge, this may be because cell membranes contribute a large fraction of biomass. At the upper edge, it may be because diffusion of substrate to the cell surface is slower per cell volume.

A secondary result has been that species identified in the literature as oligotrophic or copiotrophic are clearly separated along the dimension of maximum growth rate or rRNA operon copy number (RRN), but not along the dimensions of genome size or cell radial diameter. It is no surprise to find that identified oligotrophs are strongly separated from copiotrophs along a maximum growth rate dimension, since capacity to respond to favorable growth conditions is often a criterion people have used to label species as copiotroph versus oligotroph. Similarly, RRN is a predictor for the abundance of ribosomes produced or maintained, and low RRN is therefore connected to higher yields at the expense of slower rates, and thence to being able to sustain populations at low substrate concentrations. In the data compiled here, both maximum growth rate and RRN were strong predictors of what people have called oligotrophs versus copiotrophs.

There have been three previous reports of positive correlation across species between genome size and cell size (DeLong et al., 2010; Shuter et al., 1983; West & Brown, 2005). DeLong et al. also reported positive correlation between maximum growth rate and cell size. Differences between their results and ours arise partly from their including intracellular parasites (which contributed strongly to the small‐cell, small‐genome, slow‐growth end of their patterns) and partly from their species coverage being 10‐ to 20‐fold smaller than ours, details in Appendix B. We believe our results are more representative for this reason. In further support, Guittar et al. (2019) compiled a dataset from the literature emphasizing (but not confined to) species found in infant microbiomes. Across the 2,223 records in that dataset, correlation between genome size and cell diameter was weak at *r* ^2^ = .0031 (Guittar pers comm).

We consider three possible interpretations for the apparently independent variation found among genome size, cell size, and maximum growth rate:

- Existing measurements are too noisy
- If not‐yet‐cultured species could be included then correlation would be found
- These three traits are not the decisive ones for copiotrophy and oligotrophy; the oligotrophy to copiotrophy spectrum is not a major influence on variation across species in these traits

First, how likely is it that the measurements are so noisy that no correlation can be expected? Genome size is quite tightly characterized relative to the differences across species. For species with 10 or more records, median coefficient of variation was 3% (Nielsen et al., 2021). For maximum growth rate, fewer species are covered, the numbers are known less precisely, and variation across strains within species is hardly ever known. There is uncertainty in the actual measurement, and then also there is uncertainty as to how closely culture conditions have approached the best possible. Nevertheless, reported maximum growth rates range across more than three orders of magnitude, from less than 0.01 to more than 1 per hour. Further, maximum growth rate does increase with RRN in the genome (Figure A8; *r* ^2^ = .30). This correlation is well established, and indeed RRN has quite often been used as a surrogate or indicator for potential rate of increase (Nemergut et al., 2016; Niederdorfer et al., 2017; Roller et al., 2016; Stoddard et al., 2015; Vieira‐Silva & Rocha, 2010). Given the wide range and this established correlation, we believe the estimates for maximum growth rate do contain meaningful signal.

Cell radial diameter measurements are typically given as either a single number or a range, without specification as to what the range represents. We believe the range usually represents a sampling of individual cells within a culture, more so than different stages of the cell division cycle, different provenances within a species, or different growth conditions. We have not thought it possible to estimate any form of within‐species variation from this. Plasticity within the same genotype in response to growth versus starvation conditions is considerable (Lever et al., 2015), but measurements will nearly all have been taken under favorable growth conditions and standardized to that extent.

In summary, while there is certainly noise in the data, we do not believe it is so extreme as to obviate correlations that are there in reality.

A second possible interpretation for the apparently independent variation found among genome size, cell size, and maximum growth rate is that if not‐yet‐cultured species could be included there would be correlation. It certainly seems true that not‐yet cultured species tend toward smaller genomes (e.g., Nayfach & Pollard, 2015), and it is possible that once brought into culture, they will be found also to have smaller cells and slower potential rates of increase. While such a result would be interesting, it would not really detract from the results in Figures 1 and 2. The data available do include species that the literature regards as strong oligotrophs as well as copiotrophs, as indicated in the figures, and most ideas about the nature of oligotrophy have been developed from species brought into culture.

A third possible interpretation is that these traits are not actually among the principal traits contributing to oligotrophy versus copiotrophy. For example, Lauro et al. (2009) found that no single trait was a clear identifier of oligotrophy, and a complex multi‐trait approach was needed. We think this interpretation is the likeliest with regard to cell diameter and genome size. For the compilation we have made of species identified in the literature as oligotrophs or copiotrophs, maximum growth rate and RRN were indeed rather strong predictors. However, cell diameter and genome size were not, and were also substantially uncorrelated with maximum growth rate. These results suggest future research can usefully focus on developing stronger ecological interpretation of cell radial diameter and of genome size.

## CONFLICT OF INTEREST

None of the authors have any conflict of interest.

## AUTHOR CONTRIBUTIONS

**Mark Westoby:** Conceptualization (lead); Formal analysis (equal); Funding acquisition (equal); Methodology (equal); Project administration (lead); Visualization (equal); Writing‐original draft (lead); Writing‐review & editing (equal). **Daniel Aagren Nielsen:** Conceptualization (equal); Data curation (equal); Formal analysis (lead); Visualization (equal); Writing‐review & editing (equal). **Michael R Gillings:** Conceptualization (equal); Methodology (equal); Writing‐review & editing (equal). **Elena Litchman:** Conceptualization (equal); Writing‐review & editing (equal). **Joshua Madin:** Conceptualization (equal); Data curation (equal); Formal analysis (equal); Methodology (equal); Visualization (equal); Writing‐review & editing (equal). **Ian T Paulsen:** Conceptualization (equal); Funding acquisition (equal); Writing‐review & editing (equal). **Sasha G. Tetu:** Conceptualization (equal); Writing‐review & editing (equal).

## Figures and Tables

**FIGURE B1:**
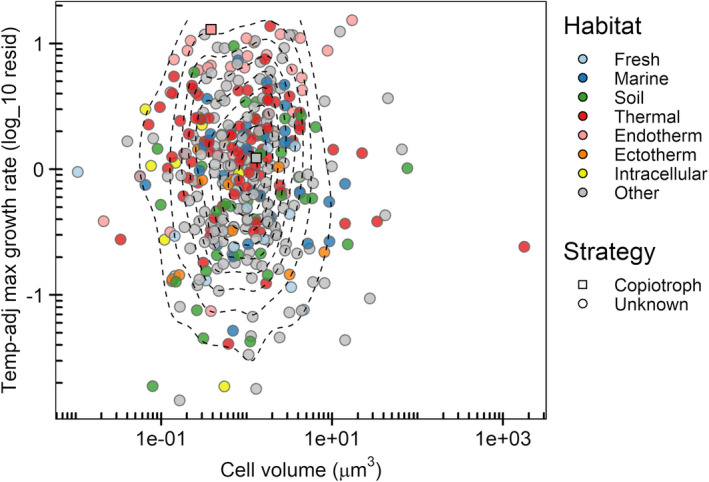
Maximum growth rate in relation to cell volume across 390 species, including intracellular, from our data. *R* ^2^ = .00043, *F*‐statistic 0.1659 on 1 and 382 *df*, *p*‐value .684

**FIGURE B2:**
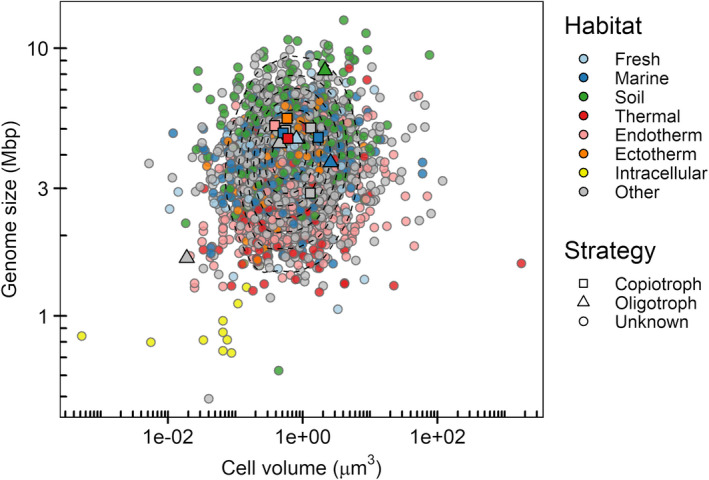
Genome size in relation to cell volume across 2,628 species, including intracellular, from our data. *R* ^2^ = .01989, *F*‐statistic 53.32 on 1 and 2,627 *df*. Leaving out the 14 intracellular species *R* ^2^ = .0119, *F*‐statistic 31.69 on 1 and 2,613 *df*

## Data Availability

Data analyzed here are drawn largely from a data paper (Madin et al., 2020) that merges multiple sources. The version used here is the product condensed to one row per species, species being defined by Genome Taxonomy Database https://gtdb.ecogenomic.org/. Code and data are at a github repo https://github.com/bacteria‐archaea‐traits/major‐dimensions/releases/tag/v1.0.0. A zipped folder containing the github repo is at https://doi.org/10.6084/m9.figshare.13610975

## APPENDIX A

### Supplementary figures and tables

**TABLE A1:** Species identified in the literature as definitely oligotroph or definitely copiotroph and indicated accordingly in the figures. Species nomenclature follows GTDB taxonomy

Species	Oligotroph or copiotroph	Habitat in brief	Reference
Gimesia maris	Oligotroph	Host	Lauro et al. (2009) and Lever et al. (2015) Table S5
Pseudoalteromonas distincta	Copiotroph	Marine	Lauro et al. (2009) and Lever et al. (2015) Table S5
Paraglaciecola atlantica	Copiotroph	Host	Lauro et al. (2009) and Lever et al. (2015) Table S5
Burkholderia cepacia	Oligotroph	Host	Tada and Inoue (2000)
Escherichia dysenteriae	Copiotroph	Host	Boutte and Crosson (2013)
Serratia marcescens_I	Copiotroph	Host	Pekkonen et al. (2013)
Photobacterium angustum	Copiotroph	Host	Lauro et al. (2009) and Lever et al. (2015) Table S5
Vibrio cholerae	Copiotroph	Host	Lauro et al. (2009) and Lever et al. (2015) Table S5
Aliivibrio fischeri	Copiotroph	Host	Lauro et al. (2009) and Lever et al. (2015) Table S5
Vibrio vulnificus	Copiotroph	Host	Lauro et al. (2009) and Lever et al. (2015) Table S5
Flavobacterium lindanitolerans	Copiotroph	NA	Lauro et al. (2009) and Lever et al. (2015) Table S5
Rhodococcus erythropolis	Oligotroph	Host	Ohhata et al. (2007)
Roseobacter denitrificans	Oligotroph	Host	Lauro et al. (2009) and Lever et al. (2015) Table S5
Novosphingobium capsulatum	Oligotroph	Host	Pekkonen et al. (2013)
Sphingomonas paucimobilis	Oligotroph	NA	Tada and Inoue (2000)
Alteromonas abrolhosensis	Copiotroph	Marine	Ivars‐Martinez et al. (2008)
Vibrio coralliirubri	Copiotroph	Host	Lauro et al. (2009) and Lever et al. (2015) Table S5
Erythrobacter_C litoralis	Oligotroph	NA	Lauro et al. (2009) and Lever et al. (2015) Table S5
Bradyrhizobium oligotrophicum	Oligotroph	Soil	Ohta and Hattori (1983)
Novosphingobium aromaticivorans	Oligotroph	NA	Lauro et al. (2009) and Lever et al. (2015) Table S5
Shewanella frigidimarina	Copiotroph	Marine	Lauro et al. (2009) and Lever et al. (2015) Table S5
Shewanella baltica	Copiotroph	Marine	Lauro et al. (2009) and Lever et al. (2015) Table S5
Ruegeria_B pomeroyi	Copiotroph	Marine	Cottrell and Kirchman (2016)
Sphingopyxis alaskensis	Oligotroph	Marine	Lauro et al. (2009) and Lever et al. (2015) Table S5
Halonatronum saccharophilum	Copiotroph	NA	Zhilina et al. (2001)
Caulobacter vibrioides_A	Oligotroph	NA	Lauro et al. (2009) and Lever et al. (2015) Table S5
Nonlabens sp000153385	Copiotroph	Marine	Lauro et al. (2009) and Lever et al. (2015) Table S5
Rhizorhabdus wittichii	Oligotroph	Host	Lauro et al. (2009) and Lever et al. (2015) Table S5
Shewanella denitrificans	Copiotroph	Marine	Lauro et al. (2009) and Lever et al. (2015) Table S5
Pelagibacter_A ubique_B	Oligotroph	Marine	(Lever et al. (2015) Table S5 citing to Cho and Giovannoni (2004); Könneke et al. (2005)).
Yoonia vestfoldensis_A	Oligotroph	Marine	Lauro et al. (2009) and Lever et al. (2015) Table S5
Rhodopirellula baltica	Oligotroph	Marine	Lauro et al. (2009) and Lever et al. (2015) Table S5
EhC01 sp000013565	Oligotroph	Marine	Lauro et al. (2009) and Lever et al. (2015) Table S5
Polaromonas sp000013865	Oligotroph	NA	Lauro et al. (2009) and Lever et al. (2015) Table S5
Sphingomonas oligophenolica	Oligotroph	Soil	Ohta et al. (2004)
Pararheinheimera texasensis	Oligotroph	Fresh	Merchant et al. (2007)
Sphingobium sp000153545	Oligotroph	Marine	Lauro et al. (2009) and Lever et al. (2015) Table S5
Nitrosopumilus maritimus	Oligotroph	NA	(Lever et al. (2015) Table S5 citing to Cho and Giovannoni (2004); Könneke et al. (2005)).
Shewanella loihica	Copiotroph	Therm	Lauro et al. (2009) and Lever et al. (2015) Table S5
BACL14 sp000168995	Oligotroph	Marine	Lauro et al. (2009) and Lever et al. (2015) Table S5
Yoonia sp000169435	Oligotroph	NA	Lauro et al. (2009) and Lever et al. (2015) Table S5

**TABLE A2:** Correlation *R* ^2^ from phylogenetic generalized least squares among the three key traits considered here, all log‐scaled. Number of phylogenetic tree nodes for each trait pair given next to the correlation

Trait	Genome size	Cell radial diameter
Temperature‐adjusted max growth rate	0.00147 (*n* = 268)	0.000192 (*n* = 219)
Genome size		0.00968 (*n* = 948)

**TABLE A3:** Coefficients ± *SE* for the model for log10 maximum growth rate fitted in Figure 2, treating each species as an independent item of evidence. Coefficients for each habitat are relative to fresh water, which is the intercept

	Coefficient ± *SE*	Probability *p*
Intercept (freshwater habitat)	−0.175 ± 0.099	.079
log10 cell radial diameter	0.242 ± 0.538	.65
(log10 cell radial diameter)^2^	−3.173 ± 0.534	5.26e−09 ***
Habitat Marine	0.190 ± 0.129	.14
Habitat Soil	−0.051 ± 0.126	.69
Habitat Thermal	0.300 ± 0.113	.0081**
Habitat Endotherm	0.616 ± 0.125	1.03e−06***
Habitat Ectotherm	−0.105 ± 0.160	.51
Habitat Other	0.096 ± 0.105	.36

**TABLE A4:** Models with successively more terms for predicting temperature‐adjusted maximum growth rate, showing multiple *R* ^2^ and associated *df*. All quantitative traits are log_10_ scaled. Asterisk instead of + indicates interactions are included. Models shown here use all data rows for which variables in that particular model are available; hence, for the simpler models, they have more degrees of freedom than in Table 2

Model	*R* ^2^	*df*
1. growth ~ cell_diam	.000456	519
2. growth ~ genome_size	.00425	618
3. growth ~cell_diam * genome_size	.0156	382
4. growth ~ cell_diam + cell_diam^2	.0538	518
5. growth ~ genome_size + genome_size^2	.00443	617
6. growth ~ habitat	.124	882
7. growth ~ cell_diam + cell_diam^2 + habitat	.154	512
8. growth ~ (cell_diam + cell_diam^2) * habitat	.174	500
9. growth ~ (cell_diam + cell_diam^2) * genome_size * habitat	.232	344

## APPENDIX B

### Previous reports of positive correlation among maximum growth rate, cell size, and genome size

Kempes et al. (2016), with precursors in (DeLong et al., 2010; Kempes et al., 2012), developed arguments about upper and lower limits of cell size in bacteria and archaea. In the course of this, they reported positive scaling of genome size and of maximum growth rate with cell volume. We have come to the view that the difference between their results and ours traces mainly to differences in the sets of species included.

Consider first the positive relationship between maximum growth rate and cell size reported by DeLong et al. (2010) (RMA slope 0.73, data in their Table S2). Although this relationship was significant, it had an *r* ^2^ of only .16 across 35 species. Further, the significance arose from inclusion of four mycoplasma species, three of which have particularly small cell sizes and slow maximum growth rates. With mycoplasmas excluded, the relationship was not significant (*r* ^2^ = .02, *df* = 31, *p* = .40). Figure B1 shows the relationship to volume across 383 species from our data, and the correlation is negligible either excluding (*r* ^2^ = .00046) or including (*r* ^2^ = .00043) species that make a living inside eukaryote cells. These intracellular species contribute only 7/383 (<2%) in our data, versus 4/35 (>10%) in DeLong et al. (2010).

Kempes et al. (2016) reported a log–log scaling slope of 0.21 between genome size and cell volume across 145 taxa. Their data were compiled from three previous reports (DeLong et al., 2010; Shuter et al., 1983; West & Brown, 2005). Kempes et al Table S1 provides the data but not the species names, and we have not been able to elicit them otherwise, except for Shuter et al who published names along with data. Consequently, we have only been able to investigate the consequences of including particular species where our coverage overlaps with Shuter et al. (1983). Across Shuter’s 49 records, *r* ^2^ was .73. One mycoplasma species with notably small genome and cell size contributed to this, but was not solely responsible. Across our dataset’s records for 12 species that also occurred in Shuter et al, the relationship was similarly positive with *r* ^2^ = .30. Across our dataset as a whole (Figure B2), the relationship was notionally positive but very much weaker (slope 0.044 ± 0.006 CI compared with 0.22 ± 0.019 for Shuter et al) even including the 14 intracellular species. Similar to the relationship between maximum growth rate and cell size, this indicates the difference between their results and ours lies mainly in coverage of species, rather than in different estimates for the same species. For our dataset (Figure B2), it can be seen that intracellular species do tend to lie toward lower left, but they are not sufficient in number to create a strong positive relationship.

In summary, our opinion is that our results indicating little to no correlation between cell size and maximum growth rate or genome size are more representative than the positive relationships reported by Kempes et al. (2016). For maximum growth rate, their positive relationship depends entirely on including mycoplasmas. For genome size, the very weak correlation with cell size reported in our results is based on 3,466 species for cell diameter or 2,628 species for cell volume compared with 145 observations in Kempes et al. (2016).

## APPENDIX C

### Temperature adjustments to maximum growth rate

Maximum growth rates are influenced by the growth temperature where they were measured. An ideal adjustment of maximum growth for temperature would express them relative to the fastest growth rates that could potentially be achieved by species that had over evolutionary time fully optimized their physiology in relation to the temperature. However, there is no consensus how this could be done.

The simplest adjustments apply a Q10 increase factor (usually 2 or 1.5) per 10°C increase in temperature to metabolic rates or growth rates. When a Q10 of 2 is applied to adjust maximum growth rates to a standard growth temperature of 37°C, many thermophiles have decidedly slow growth rates. We cannot tell whether this is biologically realistic—for example, the protein adjustments needed for high temperatures might prevent rapid metabolism—versus whether a Q10 of 2 is too steep. Thermophilic enzymes are in general stiffened to counteract the increased molecular motion associated with higher temperatures. When operating at room temperature, they typically have either lower or similar activities compared to their mesophilic homologs (Chang et al., 2020).

It is well established that Q10 itself changes with temperature. Considering soil respiration and decomposition rates, meta‐analysis showed Q10 around 4–6 at 0°C declining to 2 at around 25°C and continuing around 2 out to 50°C (Hamdi et al., 2013). A review of theoretical equations for temperature response (Noll et al., 2020) considered 19 models that are variations on Arrhenius (linear response of ln growth or metabolic rate to reciprocal of absolute temperature) from 1946 up to the present. Several of these equations have growth rates declining above some optimal temperature. If these were applied, the effect would be for species growing at 70–100°C to have their growth rates increased rather than decreased when adjusted down to 37°C. Different mechanisms are invoked by different models, but enzyme adaptation is not among them.

In absence of a consensus method for temperature‐adjusting maximum growth rates, we have adopted residuals after regression of log10 max growth rate on growth temperature. These residuals measure how much faster or slower a species grows compared with the mean at that growth temperature. The regression on temperature in deg C did not have noticeably inferior fit compared with Arrhenius regression on 1/absolute temperature (*r* ^2^ = .110 vs. .114).
